# 

**Published:** 1863-02

**Authors:** 


### CONTENTS.

### ORIGINAL CONTRIBUTIONS.

ART. III. Valedictory Address. By F. Mahla, A.M., Ph. D., Prof. of Chem. Med. Dep. Lind University, .......... 65
ART. IV. Two Successful Cases of Ovariotomy. By W. H. Byford, M.D., Professor of Obstetrics, &c., Med. Dep. Lind University, 76
ART. V. Ovarian Pregnancy. By R. B. Treat, M.D., of Janesville, Wis., .......... 86
ART. VI. Ligation of the Femoral Artery, Twenty Days after being Wounded. By R. B. Treat, M.D., and Amos S. Jones, M.D., of Janesville, Wisconsin, .......... 89
ART. VII. New Isinglass Adhesive-Plaster. By E. Andrews, M.D., Prof. of Surgery in the Chicago Medical College, .......... 93

### THE CLINIQUE.

Dropsy, and the Pathological Conditions that give Rise to it. By N. S. Davis, M.D., Attending-Physician, .......... 95

### SELECTIONS.

Camp Fever, alias Scorbutic Fever. By Alex. McBride, M.D., .......... 104
A New Solid Bandage, .......... 103
Causes of the Decay of Teeth, .......... 112

### BOOK NOTICES.

Clinical Lectures on Diseases of Women. By J. Y. Simpson, M.D., F.R.S.E., Prof. of Midwifery in the University of Edinburgh, &c. .......... 113
The Principles and Practice of Obstetrics. By Gunning S. Bedford, A.M., M.D., Prof. of Obstetrics, &c., University of New York, .......... 115

### EDITORIAL.

Medical Department of Lind University, .......... 116
Illinois State Medical Society, .......... 117
Chicago Medical Society, .......... 118
American Medical Association, .......... 118
New York Ophthalmic School and Hospital, .......... 119
College Appointments, .......... 120
Books and Pamphlets Received, .......... 121
Acknowledgement. Prof. E. Andrews, .......... 121
Tribute to Army Surgeons, .......... 122
McLean Co. Medical Society, .......... 123
Logwood as a Deodorizing Agent, .......... 123
Galvanism applied, by Aid of the Laryngoscope, to the Vocal Cords, .......... 124
Resignation of Professor Samuel Jackson, .......... 124
Contagion of Secondary Syphilis, .......... 124
The Marshall Hall Physiological Test for Strychnine, .......... 125
Personal.—Surgeon Horace Wardner, .......... 125
Medical Secrecy, .......... 126
Curara in Hydrophobia, .......... 126
Remarkable Instance of the Glancing of a Minie Ball, .......... 126
Statistics of the Globe, .......... 127

